# Electrochemical Biosensor for SARS-CoV-2 cDNA Detection Using AuPs-Modified 3D-Printed Graphene Electrodes

**DOI:** 10.3390/bios12080622

**Published:** 2022-08-10

**Authors:** Luiz R. G. Silva, Jéssica S. Stefano, Luiz O. Orzari, Laís C. Brazaca, Emanuel Carrilho, Luiz H. Marcolino-Junior, Marcio F. Bergamini, Rodrigo A. A. Munoz, Bruno C. Janegitz

**Affiliations:** 1 Department of Nature Sciences, Mathematics and Education, Federal University of São Carlos, Araras 13600-970, SP, Brazil; 2 Department of Physics, Chemistry and Mathematics, Federal University of São Carlos, Sorocaba 18052-780, SP, Brazil; 3 São Carlos Institute of Chemistry (IQSC), University of São Paulo (USP), São Carlos 13566-590, SP, Brazil; 4 National Institute of Science and Technology in Bioanalysis-INCTBio, Campinas 13083-970, SP, Brazil; 5 Chemistry Department, Laboratory of Electrochemical Sensors (LabSensE), Federal University of Paraná, Curitiba 81531-980, PR, Brazil; 6 Institute of Chemistry, Federal University of Uberlândia, Uberlândia 38400-902, MG, Brazil

**Keywords:** electrochemical (bio)sensor, 3D printed electrode, AuP modified electrode, SARS-CoV-2, creatinine

## Abstract

A low-cost and disposable graphene polylactic (G-PLA) 3D-printed electrode modified with gold particles (AuPs) was explored to detect the cDNA of SARS-CoV-2 and creatinine, a potential biomarker for COVID-19. For that, a simple, non-enzymatic electrochemical sensor, based on a Au-modified G-PLA platform was applied. The AuPs deposited on the electrode were involved in a complexation reaction with creatinine, resulting in a decrease in the analytical response, and thus providing a fast and simple electroanalytical device. Physicochemical characterizations were performed by SEM, EIS, FTIR, and cyclic voltammetry. Square wave voltammetry was employed for the creatinine detection, and the sensor presented a linear response with a detection limit of 0.016 mmol L^−1^. Finally, a biosensor for the detection of SARS-CoV-2 was developed based on the immobilization of a capture sequence of the viral cDNA upon the Au-modified 3D-printed electrode. The concentration, immobilization time, and hybridization time were evaluated in presence of the DNA target, resulting in a biosensor with rapid and low-cost analysis, capable of sensing the cDNA of the virus with a good limit of detection (0.30 µmol L^−1^), and high sensitivity (0.583 µA µmol^−1^ L). Reproducible results were obtained (RSD = 1.14%, *n* = 3), attesting to the potentiality of 3D-printed platforms for the production of biosensors.

## 1. Introduction

Versatility, design freedom, and low cost are differential characteristics of the construction of analytical systems and devices [1]. In such a context, the use of 3D printing technology is very attractive due to its capacity of converting conventional and centralized manufacturing processes into a rapid, in-lab, and customizable prototyping process, allowing the obtention of a wide variety of structures in a simple way [2]. The use of fused deposition modeling (FDM) in 3D printers provides three-dimensional objects after the deposition of thermoplastic filaments layer-by-layer, through a heated nozzle [3]. The emergence of conductive filaments allowed the use of 3D printing for the preparation of electrochemical devices. These filaments are usually composed of carbon black (CB) or graphene (G) as conductive materials and polymers such as polylactic acid (PLA) [4]. The production of 3D printed sensors based on graphene has been widely explored and is an interesting option. The unique physical and electrochemical properties of this material provide adequate characteristics to be used as electrochemical sensors. Among these properties, it can be mentioned the mechanical characteristics, increased surface area, and excellent electrochemical activity with the potential to produce highly sensitive devices [5,6,7]. Once those filaments are commercialized, the electrochemical sensors can be easily obtained. These materials are presented as promising for the development of several (bio)sensors, even though a high amount of insulating material is present, requiring pre-treatment steps previous to their use [3,8].

Although the use of 3D printed electrodes for sensing applications is relatively new and is still being improved by the exploration of different materials, these devices’ potential as platforms for the construction of biosensors has already been shown, providing interesting results [9]. For example, Muñoz and Pumera (2021) reported the production of 3D printed immunosensors based on graphene/PLA modified with gold nanoparticles for detection of the spike protein of the SARS-CoV-2 virus [10]. In the same year, Martins and co-workers developed a CB/PLA-based 3D printed immunosensor for determining Hantavirus Araucaria nucleoprotein [10]. Thus, the use of 3D-printed electrodes as a platform for biosensing of diseases is promising and has gained space for the growth and development of a new research line.

During the last decades, infectious diseases transmitted by viruses have become a significant concern worldwide [11]. The emergence of new viruses, as well as their mutability, is common. In 2019, the entire world was surprised by the beginning of what would become a new global pandemic, caused by a respiratory virus. This severe acute respiratory syndrome coronavirus 2 (SARS-CoV-2), responsible for causing coronavirus 2019 disease (COVID-19), was firstly reported in Wuhan, China [12].

As one of the most effective manners of controlling the spread of SARS-CoV-2 relies on the isolation of infected patients, there is a need to develop fast, reliable, and simple disease detection methods that can supply the demand and identify asymptomatic individuals [13]. Several works have reported the development of point-of-care devices for the diagnosis of COVID-19 [14]. However, the standard diagnosis method for SARS-CoV-2 relies on the use of polymerase chain reaction (PCR) to amplify the genetic material for the detection of the virus [15]. PCR is highly sensitive and reliable, but it presents limitations. The average time for generating results is low (24–72 h) and, the time between collection and delivery of samples is unpredictable due to the scale of the global COVID-19 pandemic [16]. Furthermore, these methods require well-trained professionals, well-equipped laboratories, and sophisticated instruments. In this context, the implementation of a low-cost, real-time detection method for the screening of SARS-CoV-2 is of high priority.

In this aspect, the use of electrochemical methods for the detection of this virus is an interesting alternative [17]. Different types of electrochemical biosensors for the detection of SARS-CoV-2 and other viruses, such as aptasensors, immunosensors, and DNA sensors, have been already reported [18,19,20,21,22,23]. Among these, the work of Li et al. [22], 2015, who developed a paper-based electrochemical DNA sensor for the detection of the hepatitis B virus can be mentioned. Ghanbari et al. [21] 2017 developed a novel electrochemical aptasensor for ultrasensitive detection of the hepatitis C virus. Regarding the detection of SARS-CoV-2, Brazaca et al. [23], in 2022, developed a low-cost immunosensor (< US$ 0.03 per device) based on gold-modified screen-printed carbon electrodes for the detection of spike (S) protein of the SARS-CoV-2 virus. However, only a few explore the use of 3D printing technology. Recently, our research group addressed the obtention of new conductive filaments for 3D printing electrochemical sensors, composed of graphite/PLA (40% *w*/*w*) as a platform for the development of a new immunosensor for the detection of SARS-CoV-2 [24]. The immunosensor was fabricated through the immobilization of SARS-CoV-2 spike protein antibodies directly at the unmodified electrode, and since the obtained surface presented the functional groups, a need for surface modification with metallic particles was eliminated. The biosensor was able to detect the spike (S1) protein of the virus with high sensitivity (0.01 µA µg^−1^ mL), enabling the detection of the spike (S1) protein of the virus in synthetic saliva samples. On the other hand, as presented in this work, the use of Au can improve the electric conductivity and increase in the surface area, promoting interesting characteristics to the applicability of the electrodes in biosensing.

The development of genosensors is appreciable clinically and from the point of view of collective health, allowing the monitoring and diagnosis of infectious diseases. These sensors are based on surface modifications with specific genetic materials that cause changes in measurable properties upon hybridization [25]. Thereby, using genosensors, the genetic material of the virus (RNA or cDNA) can be detected using electrochemical techniques for determining the hybridization rate of specific cDNA strands with their complementary probes immobilized on the surface [20,26,27]. To our knowledge, works in the literature reporting the development of 3D-printed genosensors for the detection of SARS-CoV-2 are scarce to date. Therefore, the use of 3D-printed platforms for the detection of COVID-19 has yet to be explored. In the work developed by Crevillen et al. [28] the use of 3D pen-printed electrodes (3D-PP), composed of graphene/PLA for the development of a genosensor by the adsorption of the probe (ssDNA), which targeted the N gene sequence of the virus SARS-CoV-2, was reported. The hybridization of ssDNA and viral RNA caused desorption of ssDNA on the electrode surface, and the electrochemical oxidation of the adenine present in the non-desorbed probe occurred, providing a voltammetric response. The genosensor was integrated into a PDMS microfluidic channel, providing a lab-on-a-chip system, capable to detect the RNA of SARS-CoV-2.

In addition, the detection of biomarkers for SARS-CoV-2 can be an interesting approach to improving clinical diagnosis. COVID-19 has been commonly associated with kidney damage, which can be diagnosed by elevated levels of creatinine (CNN) [29,30]. CNN is produced in the muscles from creatine, released into the bloodstream, and excreted by the kidney [31]. The measurement of CNN levels in human blood or urine is clinically essential because the levels partially reflect the state of renal and muscle function and high levels of creatinine (greater than 150.0 or 500.0 µmol L^−1^) indicate malfunction of the kidneys [31,32,33]. Values below 40.0 µmol L^−1^ are indicative of decreased muscle mass [33,34,35,36]. It is also extremely important to monitor the presence of this possible COVID-19 biomarker since it can indicate an early infection of the patient by the virus [30,37].

In this scenario, electrochemical sensors are presented as attractive analytical devices because they combine high sensitivity, simplicity of operation, and low cost [38]. In the literature, various types of enzymatic [39] and non-enzymatic [40,41] sensors are reported for the determination of CNN with different electrochemical techniques [42,43,44]. Although selective, enzyme biosensors have some limitations when compared to non-enzymatic electrodes. In this context, the high cost and denaturation, time-consuming preparation, and lack of stability can be highlighted as negative points that can be contoured when non-enzymatic sensors are applied. Thus, the development of non-enzymatic sensors for the detection of CNN is a highly viable and advantageous option [40,45,46]. However, works that explore the use of miniaturized and portable devices for CNN determination are still lacking, and the use of 3D-printed devices for the detection of CNN has not been reported in the literature until now.

In this aspect, the versatility in the production process employing the 3D printing technique [3,47,48,49], linked with the use of electrodeposited electrocatalytic metals on the electrode surface, can improve sensitivity, stability, and applicability in electroanalytical devices. The use of gold particles for electrode modification is desired, for its good conductivity, biocompatibility, and chemical and electrocatalytic properties, and for being interesting materials for the modification of electrochemical (bio)sensors [50,51,52].

Therefore, in this work, we present for the first time the development of a 3D-printed cDNA biosensor for the detection of SARS-CoV-2 in human serum and synthetic saliva using a simple, rapid, low-cost, miniaturized, and versatile graphene/PLA-based 3D- printed platform modified with Au particles. In addition, we explored the proposed platform for the signal-off sensing of the CNN biomarker in synthetic urine and human serum.

## 2. Materials and Methods

Ultrapure water from a Milli-Q Plus system (Millipore Corporation^®^, Darmstadt, Germany) was used to prepare all the aqueous solutions. Sodium chloride (NaCl) (98%, Vetec^®^, Rio de Janeiro, Brazil) was used as the supporting electrolyte in voltammetric analyses to determine CNN. The electrode modification was performed with gold(III) chloride trihydrate (99%, Sigma-Aldrich^®^, St. Louis, MO, USA) in sulfuric acid (H_2_SO_4_) (95%, Vetec^®^, Brazil). Interfering tests were performed using glucose, ascorbic acid (AA) (Sigma-Aldrich^®^, USA), uric acid (AU) (99%, Sigma-Aldrich^®^, USA), and reduced glutathione (98%, Sigma-Aldrich^®^, USA). A 10.0 mmol L^−1^ stock solution of creatinine was used as an analytical standard.

Both synthetics (urine and saliva) were prepared following protocols from the literature. Synthetic urine was produced with NaCl (98%, Vetec^®^, Brazil), potassium chloride (99%, Vetec^®^, Brazil), calcium chloride dihydrate (99%, Vetec^®^, Brazil), anhydrous sodium sulfate (99%, Vetec^®^, Brazil), monopotassium phosphate (99%, Vetec^®^, Brazil), ammonium chloride (99%, Vetec^®^, Brazil), and urea (99%, Dinamica^®^, Indaiatuba, Brazil) [53]. Synthetic saliva was prepared with sodium chloride (99%, Vetec^®^, Brazil), sodium phosphate dibasic (99%, Vetec^®^, Brazil), potassium chloride (≥99%, Vetec^®^, Brazil), potassium thiocyanate (99%, Vetec^®^, Brazil), and urea [54]. The human serum was purchased from Sigma-Aldrich^®^, USA.

For the development of the biosensor and further analysis, 2-mercaptoethanol and Tris Buffered Saline 10× were acquired (Sigma-Aldrich^®^, USA). Three DNA sequences were obtained (EXXTEND^®^, Paulinia, Brazil): the sequence capture, or probe (Thiol C6-AGATGTCTTGTGCTGCCGGTA), obtained from the gene ORF1ab, the sequence target (TAGCCGGCAGCACAAGACATCT), and the negative control sequence target (TGACTACAGAAGTGGCTTTTG).

### 2.1. Apparatus, Electrochemical Cell, and Electrodes

All electrochemical measurements were performed using a potentiostat/galvanostat PGSTAT204 Metrohm^®^ (Eco Chemie, Utrecht, The Netherlands) guided by the NOVA (version 2.1.4) software, which was also used for data acquisition and treatment. Background correction was used for voltammetric detection of CNN for better peak resolution, using the “moving average” algorithm, with window size set to 2.

A Sethi3D S3 3D printer (Campinas, Brazil), fed by both conductive PLA (doped with graphene) acquired from Black Magic^®^ 3D (New York, NY, USA) and non-conductive (PLA from Sethi3D^®^ (Campinas, Brazil)) thermoplastic filaments, were used for the manufacture of the fully 3D-printed electrochemical system, by the FDM method.

### 2.2. Morphological and Electrochemical Characterization

Scanning Electron Microscopy (SEM) Thermo Fisher Scientific model Prisma E with ColorSEM Technology and integrated energy-dispersive X-ray (EDX) spectroscopy was used to acquire SEM images. The images were obtained for G-PLA and Au/G-PLA. Infrared spectroscopy, and results were obtained by a Bruker^®^ ALPHA II Fourier transform infrared spectrometer between 4000 and 600 cm^−1^.

Electrochemical impedance spectroscopy (EIS) analyses were performed to understand the different surface effects provoked by the addition of Au on graphene working electrodes. Each study was performed by applying its specific open-circuit potential values (0.6 and 9.0 mV for G-PLA and Au/G-PLA electrodes, respectively), measured after 300 s stabilization, with a fitting χ^2^ of 0.04367 (G-PLA) and 0.0272 (Au/G-PLA).

### 2.3. Production of the G-PLA Electrode

The reference, working, and counter electrodes were manufactured with a 3D printer, with the design based on commercial SPEs (screen printed electrodes), where the working electrode consisted of a circle of 4 mm diameter, the counter electrode consisted of a semicircle of 9 mm, which was placed surrounding the working electrode, and the reference electrode consisted of a smaller semicircle to fill the space around the working electrode, as shown in Scheme S1. All three electrodes were manufactured at the tip of a 1.5 cm rectangle, responsible for connection with the potentiostat cables, which were insulated with nail polish. The electrodes were 3D printed using a commercial G-PLA filament, and the designs were drawn using Blender, and, finally, imported by Simplify3D software, which controls the 3D printer. The STL files obtained are available on the journal website. Finally, the electrodes were printed at 190 °C. After printing, the electrodes were assembled according to Scheme S1. Initially, the electrodes were fixed on support (hard plastic sheet) and over the double-sided adhesive tape, which was delimited with colorless nail polish (approximately up to half of the electrical contact) and dried for 20 min.

### 2.4. Optimization and Electrodeposition of Au

Optimization of the electrodeposition of Au on the G-PLA was conducted with a 2^2^ central composite design (CCD) with three replicates on the central point. The electrodeposition of Au was performed by applying −0.6 V constantly. The responses used in the optimization were obtained by CNN (3.0 mmol L^−1^) in NaCl (0.5 mol L^−1^) analysis by SWV with the standard operating parameters of the software (step potential (5 mV); modulation amplitude (20 mV) and frequency (15 Hz)). In this step, the variables of interest (deposition time (X_1_) and concentration of Au (X_2_)) were studied in a range of 47 to 683 s, and 0.76 to 9.25 mmol L^−1^. The obtained response surface was then used for the establishment of the best electrodeposition conditions. The experimental layout of the 2^2^ CCD with the experiments performed, variables and their respective actual and normalized levels, and CNN analytical signal response can be found in Table S1 (Supplementary Material). Therefore, the SWV parameters were set at 5 mV (step potential), 20 mV (modulation amplitude), and 10 Hz (frequency).

### 2.5. Optimization of SWV Variables for CNN

The pre-optimization of the operational parameters (step potential; modulation amplitude; and frequency) of the SWV technique was performed with a 2^3^ full factorial design at a range of 2.0 to 10 mV, 20 to 60 mV, and 6.0 to 34 Hz, respectively. This procedure evaluated the significance of the three SWV variables in a solution containing 3.0 mmol L^−1^ CNN and 0.5 mol L^−1^ NaCl. Table S2 shows the 2^3^ full factorial designs (variables and their respective actual and normalized levels, and CNN analytical signal response). The experiments in Table S2 were performed in a random order [55,56].

Subsequently, the two significant variables (step potential and frequency) were optimized by applying a 2^2^ CCD at a range of (1.0 to 7.0 mV and 6.0 to 34 Hz) in a solution of 5.0 mmol L^−1^ CNN in 0.5 mol L^−1^ NaCl. The 2^2^ CCD matrix of the optimization step can be found in Table S3.

### 2.6. Biosensor Preparation

The biosensor was prepared using the G-PLA sensor previously modified with Au by electrodeposition. For the development of the biosensor, the Au deposition time was also optimized, and the optimization was performed using univariate experiments applying −0.6 V constantly, in which the concentration was fixed at 5.0 mmol L^−1^, after the previous optimization. The deposition time varied from 100 to 300 s and the cyclic voltammetry technique was selected for the analysis employing a 1.0 mmol L^−1^ ferrocenemethanol redox probe in 0.1 mol L^−1^ KCl.

Subsequently, the Au/G-PLA was the base for the immobilization of the cDNA capture sequence by the drop-casting method, in which the immobilization time and concentration were optimized. The capture cDNA immobilization occurs because the thiol groups present on the cDNA strand bind to the gold particles present on the surface of the 3D-printed sensor, allowing the capture strand to be anchored. Initially, a solution containing 100.0 µmol L^−1^ of the capture sequence and 0.12 mmol L^−1^ 2-mercaptoethanol in 10.0 mmol L^−1^ TRIS buffer was prepared as a stock solution. All subsequent dilutions were carried out with ultra-pure water following a previous protocol from the literature [17]. The optimization method was multivariate, using the central composite design, employing a range from 1.0 to 7.0 µmol L^−1^ of capture sequence (X_1_) and hours (X_2_) for both parameters. The variables, their respective actual and normalized levels, and their analytical signal responses (current difference between the Au/G-PLA signal to the biosensor employing 1.0 mmol L^−1^ ferrocenemethanol in 0.1 mol L^−1^ KCl) can be found in Table S4 (Supplementary Material).

**Scheme 1:**
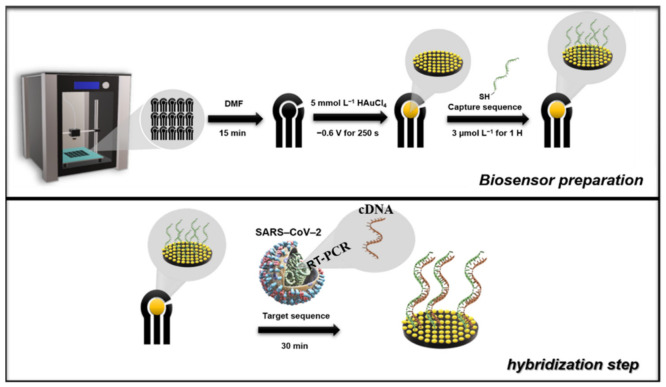
Schematic illustration of the production of the biosensor and hybridization step. The production of the biosensor consists of the printing step, chemical treatment of the surface, modification with Au (5.0 mmol L^−1^), and, finally, modification of the surface with the capture sequence (3.0 µmol L^−1^ for 1 h). The hybridization step is carried out for 30 min after adding the drop of solution containing the target sequence to the surface of the biosensor.

For analysis of the target sequence, a drop containing the biomolecule was cast on the surface of the biosensor. It is expected that the target sequence hybridizes with the capture sequence immobilized on the surface of the electrode. The hybridization time was optimized by univariate experiments, varying the time from 30 to 180 min with a concentration of 1.0 µmol L^−1^ of the target sequence. The biosensor production, as well as the hybridization step (determination of the target sequence), was present in Scheme 1, and for better comprehension of the whole process, a time-lapsed video showing the 3D printing of the electrodes and biosensor production steps and the STL files for 3D printing are available on the journal website.

## 3. Results and Discussion

### 3.1. Morphological and Electrochemical Characterization

**Figure 1:**
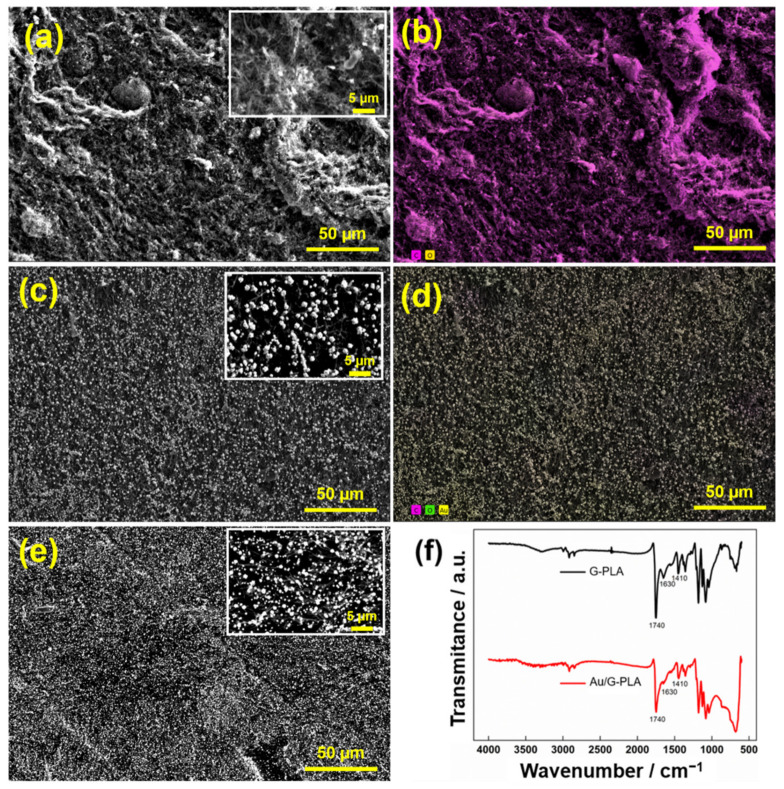
SEM images of electrode surfaces (**a**) G-PLA, (**b**) corresponding element mapping of G-PLA, (**c**) Au/G-PLA (400 s deposition), (**d**) corresponding element mapping of Au/G-PLA, (**e**) Au/G-PLA (250 s deposition), and (**f**) FT-IR spectra of G-PLA (black) and Au/G-PLA (red) were recorded within the wavenumber range of 600 to 4000 cm^−1^.

The morphologies of the G-PLA and Au/G-PLA (250 and 400 s deposition) electrodes were characterized by SEM. The FT-IR spectra of G-PLA and Au/G-PLA were recorded within the wavenumber range of 600 to 4000 cm^−1^. Figure 1 presents SEM images obtained for the proposed electrodes and FT-IR spectra.

Figure 1a presents the surface morphology obtained by SEM analysis of the G-PLA electrode. A non-uniform surface can be observed, with large surface irregularities, probably caused by the exposition of graphene material after the surface treatment. The surface images of Au/G-PLA show, as expected, gold particles (AuPs) well-distributed on the entire surface of the sensor. Furthermore, different deposition times have shown different distribution and number of AuPs on the surface. Therefore, the optimization of this parameter was necessary for each type of desired application. In addition, the element mapping in Figure 1b shows the presence of carbon on the surface and Figure 1d shows the presence of Au is predominant. On the other hand, the FTIR spectra of G-PLA present the characteristic peaks at 1410, 1630, and 1740 cm^−1^, which can be associated with C-H_2_ bonds, and C=C bonds (aromatic ring), and C=O bonds (carboxyl/carbonyl), respectively [57]. These bands are observed due to the presence of PLA on the electrode surface. After the deposition of Au on the surface of the G-PLA, the peaks related to the C=O, C=C, and C-H_2_ bonds, presented a change in the intensity (decrease), indicating coordination between the bonds and Au. Thus, the FTIR confirms that the electrodeposition of Au occurred successfully on the surface of the G-PLA [58]. In addition, the EDX spectrum is shown in Figure S1, which demonstrates the presence of carbon, oxygen, and Au on the sensor surface, with atomic percentages of 64.8, 32.1, and 3.1, respectively.

Figure S2 shows the Bode plots for both electrodes and the Nyquist diagram of these systems. The inset presents all information of interest, including the equivalent modified Randles circuits. In Figure S2a,b, we find the Bode plots for both electrodes. As it can be noted at higher frequencies, there is a slight change in the electrolyte resistance (R_s_, from 165 to 144 Ω). However, considering the applied potential difference, the results suggested that the electrodeposition of Au has no significant charge effect in the double layer formation. Both systems presented maximums at phase values lower than π/2°, which indicates an impedance controlled mainly by the resistance factor, especially after the electrodeposition of Au. In Figure S2c, the Au/G-PLA system shows a considerably lower charge transfer resistance (R_ct_) than the naked electrode. The lesser capacitive process suggests that the former could be more sensitive toward electrochemical reactions than the bare graphene electrode.

Finally, the active surface area of the electrodes was calculated for the G-PLA electrode and Au-modified G-PLA. For this, cyclic voltammetric recordings at different scan rates (20, 40, 60, 80, 100, 120, 140, 160, 200, and 250 mV s^−1^) were performed at both electrodes, using a 1.0 mmol L^−1^ ferrocenemethanol solution in 0.1 mol L^−1^ KCl. The electroactive area was calculated using the Randles–Ševčík equation:

I_p_ = 2.69 × 10^5^ *A C D*^1/2^ ***n***^3/2^ *v*^1/2^

where I_p_ is the peak current (A), *A* the electroactive area (cm^2^), *C* the concentration of the redox probe (mol L^−1^), *D* the diffusion coefficient of the redox probe (cm^2^ s^−1^), ***n*** the number of electrons involved in the reaction, and *v* the scan rate (mV s^−1^). Figure S3 shows the obtained voltammograms for G-PLA and Au/G-PLA with the respective plot of current response in function of *v*^1/2^. The values obtained for G-PLA and Au/G-PLA were 0.16 and 0.22 cm^2^, respectively, indicating that the deposition of Au provides an increase in the active area. This result is in agreement with the reduction in the R_ct_ values observed in EIS studies.

### 3.2. 3D Printed Au/G-PLA Sensor for the Detection of CNN

#### 3.2.1. Cyclic Voltammetry

**Figure 2:**
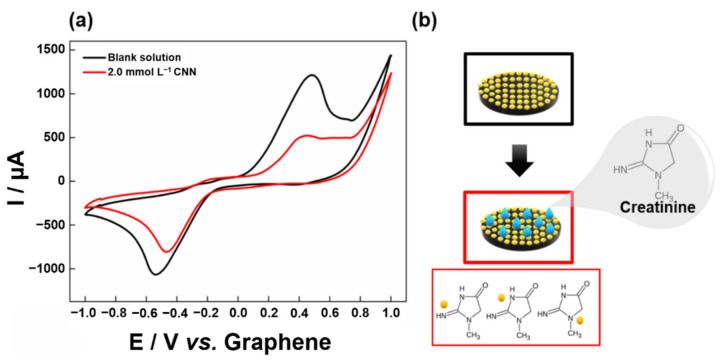
(**a**) Cyclic voltammograms of blank solution (black line) and (red line) 2.0 mmol L^−1^ CNN using Au/G-PLA electrode. Supporting electrolyte: 0.5 mol L^−1^ NaCl. Scan rate: 100 mV s^−1^, (**b**) Schematic illustration of the interaction of creatinine with AuPs.

The electrochemical response of the Au/G-PLA was evaluated using cyclic voltammetry (CV) in the presence and absence of 2.0 mmol L^−1^ CNN in 0.5 mol L^−1^ NaCl. Figure 2 shows the obtained cyclic voltammograms.

In Figure 2a, it is possible to observe that the Au/G-PLA electrode presented a typical redox process profile in cyclic voltammetry, with cathodic (−0.5 V) and anodic (0.4 V) peaks corresponding to the Au present on the electrode surface. In the presence of 2.0 mmol L^−1^ CNN, the Au current signal decays significantly, demonstrating that the interaction between the Au present at the surface of the sensor and CNN occurs successfully (Figure 2b). According to the literature, CNN contains three nitrogen groups in its structure, and the Au present on the electrode surface can bind to electron-rich nitrogen compounds through the interaction between the N and Au atoms [59,60]. Furthermore, it is noteworthy to observe that the primary amines are generally used to modify the surface of Au electrodes (mainly AuPs), and the nitrogen ring of hybrid aromatics exhibits a stronger binding affinity for Au metals [61,62,63]. Therefore, the electrochemical determination of CNN is possible by using the proposed electrode.

#### 3.2.2. Optimization and Electrodeposition of Au

**Figure 3:**
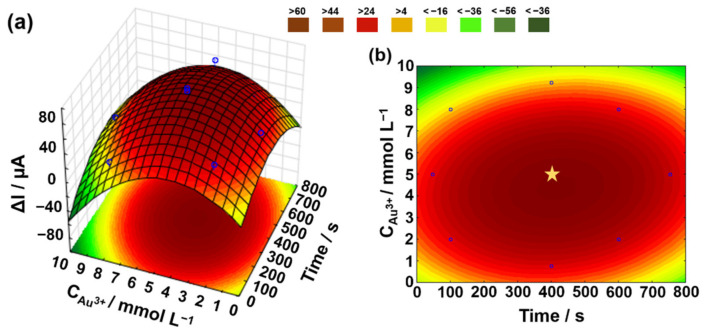
(**a**) Surface response and (**b**) level curve obtained for the optimization of the variables: Au^3+^ concentration (mmol L^−1^) and electrodeposition time (s) as a function of the current in the presence of 3.0 mmol L^−1^ CNN.

Thus, an optimization of the electrodeposition of Au onto the 3D-printed G-PLA sensor was performed based on the highest current response for CNN (difference between Au/G-PLA signal in the absence and presence of CNN), and the concentration and deposition time parameters were studied. From the responses presented in the CCD matrix (Table S1), it was possible to construct the response surface and the level curve (Figure 3a,b) to obtain the best conditions for the electrodeposition, reaching the optimized Au/G-PLA for CNN determination.

Analyzing Figure 3, the region of “maximum” current was identified (marked by the star in Figure 3b), showing a stabilized region with an optimal working interval for the variables involved. The optimal values chosen for the development of the Au/G-PLA for CNN determination were 5.0 mmol L^−1^ and 400 s for Au concentration and electrodeposition time, respectively.

#### 3.2.3. Optimization of SWV Variables

Based on the optimized conditions for Au electrodeposition, the screening of SWV variables (step potential, modulation amplitude, and frequency) was performed by a complete factorial design (2^3^). The calculated effects (step potential (X_1_), modulation amplitude (X_2_), and frequency (X_3_)) of the responses obtained in Table S2 can be seen in Table S5.

The calculated effect of the modulation amplitude variable (X_2_) was considered insignificant, even though it did not pass through the “zero” value in the confidence interval. However, the calculated effect of this parameter presented a value close to the value of the calculated third-order effect (X_123_), which in turn is considered negligible [55,56,64]. Thus, the modulation amplitude variable was set at a value of 20 mV (central point), and the other two variables were selected and studied in CCD.

By using the responses obtained and presented in Table S3, we generate the model and the construction of the response surface and the level curve, illustrating the behavior of the peak current in the presence of CNN concerning frequency × step potential. Figure S4 shows the response surface and the level curve obtained. Analyzing Figure S4, the overlapping region for maximum ΔI of CNN was identified. Thus, the selected optimal values are 5.0 mV and 10 Hz for step potential and frequency, respectively.

#### 3.2.4. Analytical Curve

**Figure 4:**
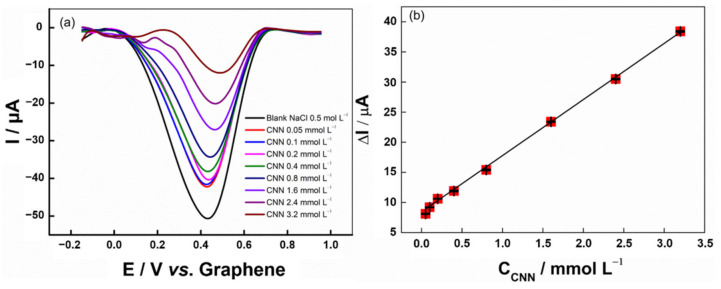
(**a**) Square wave voltammograms for the addition of eight CNN concentrations (0.05 to 3.2 mmol L^−1^) in 0.5 mol L^−1^ NaCl. SWV parameters: −5.0 mV (step potential); 20 mV (modulation amplitude); 10 Hz (frequency), (**b**) The analytical curve was obtained from the variation of I_peak_ as a function of CNN concentration.

From the previously optimized conditions of the SWV, an analytical curve for CNN was constructed in a concentration range between 0.050 and 3.2 mmol L^−1^ (Figure 4).

From the generated curve, the coefficient of determination (R^2^) obtained was 0.998 in the concentration range from 0.05 to 3.2 mmol L^−1^, which provided the equation ΔI (µA) = 8.46 + 9.318 × C_CNN_ (mmol L^−1^). The intra-electrode (*n* = 3) and intra-day (*n* = 3) precision (%RSD) values were 4.1% and 3.7%, respectively, obtained for measurements using a concentration of 0.1 mmol L^−1^ CNN. The limits of detection (LOD) and quantification (LOQ) were calculated according to the formulas LOD = (3.3 × SD_intercept_)/b and LOQ = 3 × LOD, where SD_intercpt_ is the standard deviation of three analytical curves made with the presented linear range and b is the sensitivity of the obtained calibration curve obtained from the average value of the triplicates. LOQ and LOD values were estimated to be 0.05 and 0.02 mmol L^−1^, respectively. The LOD found for the proposed procedure can determine CNN at levels low enough to indicate health problems (>150 µmol L^−1^), such as severe renal impairment, ultimately leading to dialysis or transplantation [33,34,35,36].

Table S6 presents a comparison between the data obtained from the proposed 3D-printed electrode and other sensors based on the non-enzymatic and enzymatic (biosensors) platforms for CNN detection. It can be observed that the compared works present similar linear ranges as well as LODs. However, some of the published works require the association of enzymes for the detection of CNN or more complex modifications, making them more laborious and more expensive than the proposed 3D-printed electrode. The good analytical performance of the 3D-printed electrode makes its application attractive, since large-scale production is possible, at a relatively low cost. The proposed electrode is simple to prepare and provides fast analysis.

#### 3.2.5. Interference and Recovery Test

An interference study was also accomplished to evaluate the interference caused by other species in the SWV signal of 0.7 mmol L^−1^ CNN. The study was performed by recording SWVs in the presence of glucose, AA, AU, and reduced glutathione, in the ratio of 1:5 (CNN: interferent). Table S7 shows the analytical response obtained for this study. It is possible to observe that the current response of the CNN obtained in the presence of the interferers varied from 94.70 to 109.9%. Therefore, no significant changes in the analytical signal of CNN were observed. In addition, the results are in agreement with those presented by Fava et al. 2020 [40].

An analytical recovery test verified the precision of the procedure using SWV associated with Au/G-PLA and the possibility of interference from the matrix (synthetic urine and human serum). For this step, the synthetic urine and human serum samples were enriched with three different concentrations of CNN (0.10; 0.80; and 2.10 mmol L^−1^). The recovery values are presented in Figure S5. In Figure S5, it is possible to observe that the developed protocol provided satisfactory analytical performance for CNN sensing since adequate recovery results (in the range of 98.0 to 103.0% and 95.0 to 105.0% in urine synthetic and human serum, respectively) were achieved.

### 3.3. Biosensor for COVID-19

#### 3.3.1. Biosensor Production

In the production of the biosensor, a time of 400 s with 5.0 mmol L^−1^ gold solution was used, for which the analytical signal of ferrocenemethanol (probe for the analysis of the biosensor) did not present a defined analytical signal (peak current of oxidation and reduction). Thus, the gold deposition was again optimized in function of the best analytical signal using ferrocenemethanol as a redox probe. As the gold concentration used previously proved to be an ideal condition for this purpose, only the deposition time was optimized from 100 to 300 s. The analysis was performed by cyclic voltammetry in the presence of 1.0 mmol L^−1^ ferrocenemethanol in 0.1 mol L^−1^ KCl with a scan rate of 100 mV s^−1^. The voltammograms obtained can be seen in Figure S6. The time of 250 s for deposition of Au in the biosensor development was chosen as ideal since it presents the highest analytical signal.

#### 3.3.2. Voltammetric Profile of the Proposed Biosensor

**Figure 5:**
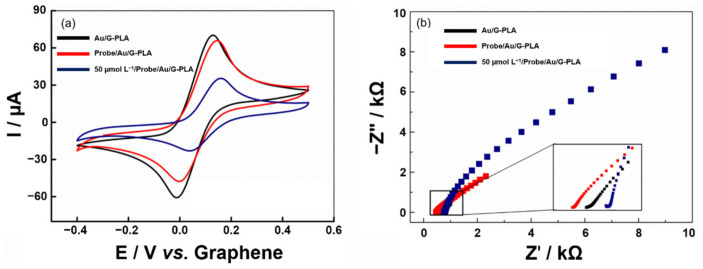
(**a**) Cyclic voltammograms and (**b**) Nyquist plots for each stage of sensor modification and after hybridization, (black line) Au/G-PLA, (red line) Probe/Au/G-PLA, and (blue line) 50.0 µmol L^−1^ target sequence. All analyses were conducted using 1.0 mmol L^−1^ ferrocenemethanol in 0.1 mol L^−1^ KCl; CVs were carried out with a scan rate of 50 mV s^−1^.

Cyclic voltammetry and EIS measurements were performed before and after the immobilization of a capture sequence (3.0 µmol L^−1^ for 1 h) on the surface of the Au/G-PLA using a 1.0 mmol L^−1^ ferrocenemethanol solution in 0.1 mol L^−1^ KCl as the supporting electrolyte. The voltammograms obtained and Nyquist plots can be seen in Figure 5.

Figure 5a presents the oxidation and reduction peaks of the redox probe ferrocenemethanol on Au/G-PLA, biosensor based on Au/G-PLA (Probe/Au/G-PLA), and biosensor in the presence of 50.0 µmol L^−1^ of the target (Target/Probe/Au/G-PLA). The peak currents obtained were approximately 65.0 and −55.0 µA for oxidation and reduction of the mediator, respectively, on Au/G-PLA. The developed biosensor presented 62.0 and −47.0 µA, respectively, showing a slight decrease in the current response after immobilization of the capture sequence on the electrode surface. After hybridization with 50.0 µmol L^−1^ of the target sequence (cDNA) of the SARS-CoV-2 virus, the current peak values decreased considerably (decay of approximately 55% in the analytical signal) due to a partial blockage of the electrode by the deposited biological material. Similar behavior can be also observed by the EIS measurements (Figure 5b), and the fits were performed using a Randles circuit. An increase in R_ct_ values after the biological material is deposited on the sensor surface. The Au/G-PLA sensor presented an R_ct_ of 200 Ω, and from the biosensor of 1.04 kΩ and after hybridization with 50.0 µmol L^−1^ target sequence, an increase to 8.76 kΩ was registered. In addition, the Bode plots can be found in Figure S7. It can be noticed that the same behavior observed in the CV studies and Nyquist plots occurred. The electrochemical impedance spectra increase whereas changes occur on the surface of the 3D-printed sensor, mainly in the frequency range between 0.1 and 10 Hz, which is the region governed by changes in the electrical double layer. Such behavior was expected since the binding between the capture cDNA and the sensor surface and the hybridization between the capture cDNA and the target of interest occurs [65]. Therefore, it can be inferred that the biosensor was successfully assembled and it responds to the target sequence, attesting to the occurrence of the hybridization process.

#### 3.3.3. Optimization of Biosensor Parameters

The steps for the fabrication of the biosensor (immobilization of capture sequence and hybridization) were optimized to obtain a wide linear range and a low LOD. Thus, initially, the concentration and immobilization time of the capture sequence were optimized using the CV technique considering the difference between the I_pa_ obtained from Au/G-PLA to Probe/Au/G-PLA in the presence of 1.0 mmol L^−1^ ferrocenemethanol in KCl 0.1 mol L^−1^. From the responses presented in the CCD matrix (Table S4), it was possible to construct the response surface and the level curve (Figure S8) to obtain the best conditions for the modification of the biosensor. In Figure S8, the region of maximum current was identified, which was marked by the “star”, showing a stabilized region with an optimal working interval for the variables involved. The optimal values chosen for the biosensor were 3.0 µmol L^−1^ and 1 h for the concentration of the capture sequence and deposition time, respectively.

Under optimal conditions, the hybridization time was then investigated. For this purpose, the electrodes were incubated in a target sequence concentration of 1.0 µmol L^−1^ was incubated in periods ranging from 30 to 180 min, with the analysis being carried out immediately after the stipulated time. The obtained results can be seen in Figure S9, in which a significant decay in the response can be observed at 30 and 60 min. However, from 60 min onwards, a lower decrease in the ΔI_pa_ is observed, demonstrating that hybridization times longer than 60 min are not effective. Therefore, 30 min was chosen as the optimal hybridization time for further studies.

#### 3.3.4. Analytical Performance of the Signal-Off Voltammetric Biosensor

**Figure 6:**
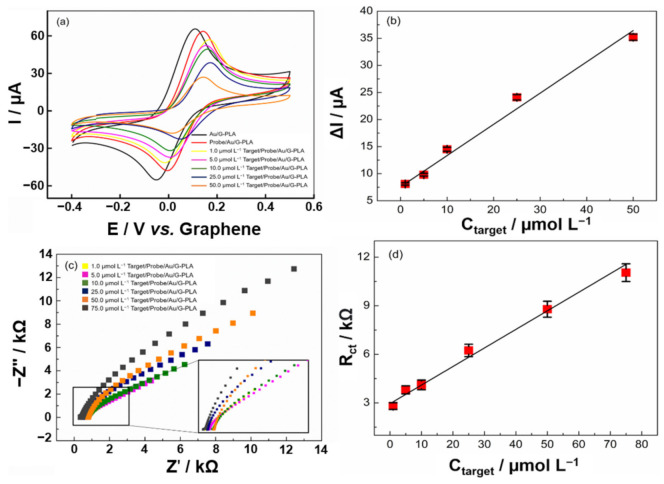
Biosensor calibration curve employing (**a**) CV and (**c**) EIS techniques, in the range of 1.0 to 50.0 µmol L^−1^ and 1.0 to 75.0 µmol L^−1^ target, respectively. (**b**) CV calibration curves were obtained between the difference of the biosensor analytical signal in the absence and presence of the target sequence employing 1.0 mmol L^−1^ ferrocenemethanol in 0.1 mol L^−1^ KCl. (**d**) EIS calibration curve was constructed from the obtained R_ct_ values.

The application of the biosensor to detect the target sequence of the SARS-CoV-2 virus was studied based on a signal-off strategy by EIS and CV techniques in a potential range from −0.4 to 0.5 V (vs. Graphene). The biosensor was tested in the presence of different concentrations of the target sequence in a range of 1.0 to 50.0 and 1.0 to 75.0 µmol L^−1^, for CV and EIS, respectively. The anodic peak currents were measured in the presence of 1.0 mmol L^−1^ ferrocenemethanol and 0.1 mol L^−1^ KCl. For each addition of the target sequence target in different concentrations on the surface of the biosensor, the anodic current response for ferrocenemethanol decreased, which indicated that the biosensor responded to different concentrations of the virus cDNA. With this, an analytical curve was obtained by the different techniques (CV and EIS). The results obtained can be seen in Figure 6.

The analytical curve obtained by the CV technique presented a linear behavior, with an R^2^ value of 0.982 in the range of 1.0 to 50.0 µmol L^−1^. Higher concentrations of the target presented a surface saturation, and no variations in the current response were observed. The equation obtained by CV was I (µA) = 0.583 × C_target_ (µmol L^−1^) − 8.536, with a sensitivity of 0.583 µA µmol^−1^ L. The detection and quantification limits were calculated as 0.30 and 0.95 µmol L^−1^, respectively. The responses obtained by EIS provided a linear behavior, with an R^2^ value of 0.987 in the range of 1.0 to 75.0 µmol L^−1^ (Nyquist plots fit the Randles circuit), and at higher concentrations, a similar behavior to that obtained by CV was observed. The equation obtained by EIS was R_ct_ (kΩ) = 0.115 × C_target_ (µmol L^−1^) + 2.977, with a sensitivity of 0.115 kΩ µmol^−1^ L. The detection and quantification limit values were found to be 0.31 and 0.93 µmol L^−1^, respectively. The reproducibility and repeatability of the biosensor were calculated using the CV technique (Figure S10), and RSDs of 1.16% (*n* = 3; 5.0 µmol L^−1^), and 1.14% (*n* = 20; 5.0 µmol L^−1^) were obtained, respectively.

It is important to mention that for the application of the biosensor in the detection of the SARS-CoV-2 in a real scenario, some operational steps must be taken into account. Such steps are (1) sample collection; (2) RNA extraction; (3) conversion and amplification of the genetic material by RT-PCR; and (4) analysis of the sample with the developed biosensor. However, steps 1–3 are the same for the current PCR-based SARS-CoV-2 detection protocol, with the biosensor not being commonly replaced by gel electrophoresis. Therefore, the biosensor is an alternative to gel electrophoresis, maintaining the same initial steps without adding operational steps and maintaining the same initial protocol that is already widely used [66,67]. It is noteworthy to mention that those steps are not exclusive to this work, and the electrochemical biosensors based on the detection of cDNA are susceptible to the same procedure [11,68,69,70].

In addition, considering a sample with a concentration of genetic material equal to the LOD of the most efficient PCR kit on the market (100 copies/mL), approximately 40 PCR cycles would be required to amplify the cDNA to reach the LOD of the biosensor developed here [66,67]. This shows that, even though the amplification is necessary, the method is viable, since 40 cycles take around 45 min to be completed.

In the literature, some works report the use of DNA/RNA for modifications of electrochemical sensors applied for the detection of the SARS-CoV-2 [28,71,72]. Table S8 presents a comparison between the data obtained from the proposed genosensor with others presented in the literature for the detection of SARS-CoV-2. The most reported sensors are screen-printed electrodes [71,73]. However, gold electrode [72], flex-printed circuit board (graphene-based) [74], platinum/titanium interdigitated electrodes on a glass substrate [16], in addition to one 3D printed sensor obtained using a 3D printing pen [28] can also be found in the literature. Among these works, the most varied stages of production of biosensors can be observed, reaching times greater than 3 h. Furthermore, LOD values ranging from 33.0 fmol L^−1^ to 0.1 µmol L^−1^ are observed. However, it is worth mentioning that some studies use high-cost materials, such as platinum, titanium, and gold, especially when compared to the materials used in the present work (mainly graphene, PLA, and gold salt). In addition, the use of 3D printing is poorly explored, with only one work using a 3D printing pen in conjunction with an ssDNA probe that targeted the N gene sequence of SARS-CoV-2. Therefore, the present work presents a new approach to 3D printing that allows the fast and automated production of miniaturized sensors, reducing operational costs and promoting easy-operation, and for the development of a genosensor for cDNA of SARS-CoV-2.

#### 3.3.5. Interfering Study

**Figure 7:**
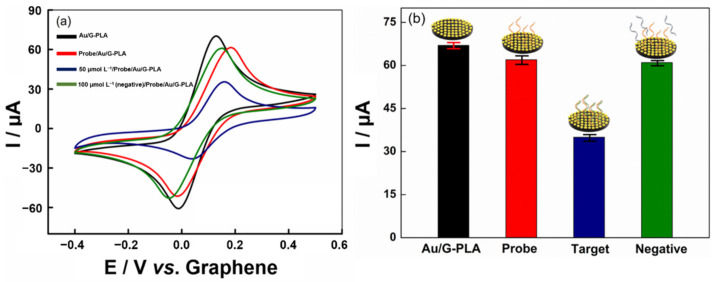
(**a**) Comparison between target sequences positive (50 µmol L^−1^) and negative (100 µmol L^−1^). Cyclic voltammograms obtained in the presence of 1.0 mmol L^−1^ ferrocenemethanol in 0.1 mol L^−1^ KCl. The scan rate was 100 mV s^−1^, (**b**) Bar column plot.

Finally, the biosensor was evaluated in the presence of a negative target (Influenza A DNA sequence) to observe the selectivity of the method. Figure 7 presents the obtained cyclic voltammograms.

As expected, the negative control DNA sequence does not show any significant change in the analytical signal relative to the biosensor signal. On the other hand, the addition of 50.0 µmol L^−1^ of the target sequence caused a sharp drop in peak current (approximately 50%), as the target sequences hybridized with the capture sequence (probe). This is a higher rate than negative sequences control, as negative sequences control does not have the necessary specificity for hybridization to occur. This behavior is attributed to its complementarity with the capture sequence. Therefore, a DNA sequence from the virus can be detected with good specificity. Furthermore, the influenza virus provides similar symptoms in an infected individual to SARS-CoV-2; thus, the DNA of this virus was chosen to be used as a negative sequence.

Finally, to demonstrate the biosensor and the sensor’s applicability, the analysis of Target was performed on synthetic saliva and human serum by recovery tests from the fortification of samples with three different concentrations of Target (1.0, 25.0, and 50.0 µmol L^−1^). The analytical responses were obtained after 30 min of incubation of the samples on the surface of the 3D-printed biosensor and the responses of the recovery values (expressed in %) can be seen in Figure S11. The recovery values obtained ranged from 96.0 to 102.0% for synthetic saliva and 97.0 to 101.0% for human serum. Therefore, the proposed biosensor proves to be suitable for sample analysis, having good potential for determining the characteristics of the SARS-CoV-2 virus, simply, quickly, and using low-cost materials.

## 4. Conclusions

Herein, we presented the development of Au-modified 3D-printed (bio)sensors with great potential for the detection of SARS-CoV-2, the virus responsible for COVID-19, and a possible biomarker for the disease, the CNN. The detection strategy employed for the construction of the analytical devices was based on the signal-off response for the Au metal present on the surface of a 3D-printed electrode, after complexation with CNN for the sensor, and after interaction with the complementary DNA strand of the virus (SARS-CoV-2) immobilized on the Au sensor surface with a target sequence (SARS-CoV-2 virus complementary DNA strand) for the biosensor. The CNN detection was successfully performed, enabling the detection of low concentrations of this analyte (LOD = 0.016 mmol L−1) with high sensitivity (9.318 µA mmol^−1^ L) in synthetic urine and human serum samples, providing adequate recovery values, which ranged from 94.0 to 110.0%. The immobilization of biologic material on the developed platform provided the fabrication of a rapid biosensor for SARS-CoV-2 detection, demanding only a 30-min hybridization time, with a low LOD (0.30 µmol L^−1^) and satisfactory sensitivity (0.583 µA µmol−1 L) in synthetic saliva and human serum samples, providing adequate recovery values, which ranged from 95.0 to 105.0%. High precision in the fabrication of the biosensors was obtained (RSD = 1.16%) and reproducible measurements were observed (RSD < 1.16%) with no interference of the Influenza A virus genetic material. Therefore, the detection of SARS-CoV-2 was performed using a simple, versatile platform, employing 3D printing technology. Even taking into account the need for amplification of cDNA by PCR, this step can still be considered fast, as previously discussed, bringing feasibility and potential for application in a real scenario.

Furthermore, the development of a rapid electrochemical test for COVID-19, combined with the determination of a biomarker would provide more informative results, such as the aggressiveness of the disease, allowing for better management and treatment in an infected population. Finally, the 3D-printed platform has the advantage of miniaturization, easy manufacturing, relatively low cost, and easy handling.

## 5. Patents

The present work generated a patent entitled “3D-printed electrochemical biosensor for COVID-19 virus detection” with Order Number: BR 10 2021 018602 0 on the date of 17 September 2021.

## Data Availability

Not applicable.

## Supplementary Materials

The following supporting information can be downloaded at: https://www.mdpi.com/article/10.3390/bios12080622/s1: Table S1. CCD planning matrix, real and normalized levels, and response obtained for Au/G-PLA. Table S2. Full factorial design matrix (2^3^) and respective results obtained for CNN. Table S3. CCD planning matrix, real and normalized levels and response for CNN. Table S4. CCD planning matrix, real and normalized levels and response for SARS-CoV-2. Table S5. Result of the effects calculated for planning 2^3^. Table S6. Electrodes found in the literature for creatinine determination. Table S7. Effect of possible interference especies on the determination of CNN. Table S8. Comparison between the proposed genosensor characteristics and works from the literature. Scheme S1. Schematic illustration of the 3D-printed electrodes assembly. Fixation on the hard plastic sheet support and delimitation of the area with colorless nail polish. Figure S1. EDX spectrum for Au/G-PLA. The scale bar corresponds to 50 µm. Figure S2. Impedance analyses of the G-PLA and Au/G-PLA electrodes. (a) impedance magnitude and frequency correlation of the G-PLA (black line) and Au/G-PLA (red line), (b) phase shift and frequency correlation of the G-PLA (black line) and Au/G-PLA (red line), (c) Nyquist diagrams of the G-PLA (black line) and Au/G-PLA (red line). Parameters: EG-PLA = 0.6 mV; EAu/G-PLA = 9.0 mV. Inset shows the respective equivalent circuits. Figure S3. Cyclic voltammograms obtained for 1.0 mmol L^−1^ ferrocenemethanol in 0.1 mol L^−1^ KCl varying the scan rate in 20, 40, 60, 80, 100, 120, 140, 160, 180, and 200 mV s^−1^ using (a) G-PLA and (c) Au/G-PLA and the respective plot of current response in function of *v*^1/2^ for (b) G-PLA and (d) Au/G-PLA. Figure S4. (a) Surface response and (b) level curve obtained for the optimization of the variables: step potential and frequency as a function of the current in the presence of 3.0 mmol L^−1^ CNN. Figure S5. Recovery test performed from fortification of samples of (a) synthetic urine and (b) human serum with three known concentrations of CNN (0.1, 0.8, and 2.1 mmol L^−1^). Figure S6. Cyclic voltammograms obtained after different gold deposition times on the G-PLA sensor. (a) (black line) 100 s, (red line) 150 s, (blue line) 200, (pink line) 250, and (green line) 300. All analyzes were carried out using 1.0 mmol L^−1^ ferrocenemethanol in 0.1 mol L^−1^ KCl with a scan rate of 50 mV s^−1^. Figure S7. Impedance analysis of the (black line) Au/G-PLA, (red line) Probe/Au/G-PLA, and (blue line) 50.0 µmol L-1 Target/Probe/Au/G-PLA. (a) impedance magnitude and frequency correlation. (b) phase shift and frequency correlation. Figure S8. (a) Surface response and (b) level curve obtained for the optimization of the variables: concentration (µmol L^−1^) and time (h) as a function of the current difference between the analytical signal of the sensor and the biosensor in presence of 1.0 mmol L^−1^ fer-rocenemethanol in 0.1 mol L^−1^ KCl. Figure S9. Responses obtained for different times (min) of hybridization of the target sequence with the biosensor. The response values were obtained as a function of the difference in the signal obtained by CV between the biosensor in the absence and the presence of 1.0 µmol L^−1^ of target sequence using 1 mmol L^−1^ ferrocenemethanol in 0.1 mol L^−1^ KCl. CVs were carried out with scan rate of 50 mV s^−1^. Figure S10. Cyclic voltammograms for (a) biosensor reproducibility (*n* = 3) and (b) repeatability (*n* = 20) in the presence of 1.0 mmol L^−1^ ferrocenemethanol in 0.1 mol L^−1^ KCl. Scan rate of 100 mV s^−1^. Figure S11. Recovery test performed from fortification of samples (a) synthetic urine and (b) human serum with three concentrations of Target (1.0, 25.0, and 50.0 µmol L^−1^). Video S1: Biosensor preparation. Refs [75,76,77] are cited in Supplementary Materials.
