# Supplementary material for: Electrochemical Biosensor for SARS-CoV-2 cDNA Detection Using AuPs-Modified 3D-Printed Graphene Electrodes
Source: Biosensors (Basel). 2022 Aug 10;12(8):622. doi: 10.3390/bios12080622 (PMC9405530; doi:10.3390/bios12080622)
Supplement: Supplementary file 1 [file biosensors-12-00622-s001.zip › Supporting material.pdf]

biosensors

MDPI

Supplementary material

Electrochemical biosensor for SARS-CoV-2 cDNA detection using AuPs modified 3D printed graphene electrodes

Luiz Silva [1,2], Jéssica Stefano [1], Luiz Orzari [1,2], Laís Brazaca [3,4], Emanuel Carrilho [3,4], Luiz Marcolino-Junior [5], Marcio Bergamini [5], Rodrigo Munoz [4,6] and Bruno Janegitz [1,*]

[1] Department of Nature Sciences, Mathematics and Education, Federal University of São Carlos, 13600-970, Araras, São Paulo, Brazil
[2] Department of physics, Chemistry and Mathematics, Federal University of São Carlos, 18052-780, Sorocaba, São Paulo, Brazil
[3] São Carlos Institute of Chemistry (IQSC), University of São Paulo (USP), 13566-590 São Carlos, SP, Brazil
[4] National Institute of Science and Technology in Bioanalysis-INCTBio, 13083-970, Campinas, São Paulo, Brazil
[5] Chemistry Department, Laboratory of Electrochemical Sensors (LabSensE), Federal University of Paraná, 81531-980 Curitiba, Paraná, Brazil
[6] Institute of Chemistry, Federal University of Uberlândia, 38400-902, Uberlândia, Minas Gerais, Brazil
* Correspondence: brunocj@ufscar.br

Table S1. CCD planning matrix, real and normalized levels, and response obtained for Au/G-PLA.

EXP	M	X_1	X_2	X_1^2	X_2^2	X_1X_2	ΔI_{CNN} (µA)
1	1	−1	−1	1	1	1	2.3
2	1	1	−1	1	1	−1	50
3	1	−1	1	1	1	−1	11
4	1	1	1	1	1	1	13
5	1	−1.4142	0	2	0	0	28
6	1	1.4142	0	2	0	0	1.4
7	1	0	−1.4142	0	2	0	33
8	1	0	1.4142	0	2	0	49
9	1	0	0	0	0	0	46
10	1	0	0	0	0	0	43
11	1	0	0	0	0	0	44

Table S2. Full factorial design matrix (2^3) and respective results obtained for CNN.

EXP	M	X_1	X_2	X_3	X_1X_2	X_1X_3	X_2X_3	$X_1X_2X_3$	CNN (µA)
1	1	−1	−1	−1	1	1	1	−1	63
2	1	1	−1	−1	−1	−1	1	1	8.3
3	1	−1	1	−1	−1	1	−1	1	46
4	1	1	1	−1	1	−1	−1	−1	8.8
5	1	−1	−1	1	1	−1	−1	1	8.5
6	1	1	−1	1	−1	1	−1	−1	8.5
7	1	−1	1	1	−1	−1	1	−1	7.5
8	1	1	1	1	1	1	1	1	8.6

Table S3. CCD planning matrix, real and normalized levels, and response for CNN.

EXP	M	X_1	X_2	X_1^2	X_2^2	X_1X_2	CNN (µA)
1	1	−1	−1	1	1	1	49
2	1	1	−1	1	1	−1	5.1
3	1	−1	1	1	1	−1	1.6
4	1	1	1	1	1	1	3.9
5	1	−1.4142	0	2	0	0	8.4
6	1	1.4142	0	2	0	0	31
7	1	0	−1.4142	0	2	0	1.4
8	1	0	1.4142	0	2	0	8.1
9	1	0	0	0	0	0	32
10	1	0	0	0	0	0	33
11	1	0	0	0	0	0	34

Table S4. CCD planning matrix, real and normalized levels, and response for SARS-CoV-2.

EXP	M	X_1	X_2	X_1^2	X_2^2	X_1X_2	ΔI_{probe} (µA)
1	1	−1	−1	1	1	1	2.6
2	1	1	−1	1	1	−1	1
3	1	−1	1	1	1	−1	2.1
4	1	1	1	1	1	1	1.2
5	1	−1.4142	0	2	0	0	2.8
6	1	1.4142	0	2	0	0	1.1
7	1	0	−1.4142	0	2	0	1.7
8	1	0	1.4142	0	2	0	1.1
9	1	0	0	0	0	0	1.8
10	1	0	0	0	0	0	1.8
11	1	0	0	0	0	0	1.7

Table S5. Result of the effects calculated for planning 2^3.

Variables	Effects	Variables	Effects
X_1	–22.70	–22.25	–23.15
X_2	–4.350	–3.900	–4.800
X_3	–23.25	–22.80	–23.70
X_1X_2	4.650	5.100	4.200
X_1X_3	23.25	23.70	22.80
X_2X_3	3.900	4.350	3.450
$X_1X_2X_3$	–4.100	–3.650	–4.550

Table S6. Electrodes found in the literature for creatinine determination.

Electrode	Technique	Linear range (mmol L^{-1})	LOD (mmol L^{-1})	Ref.
CdSe QDs	DPV	0.44 to 8.84	0.230	[1]
Transducer	Chroamperometry	0.05 to 1.00	0.0270	[2]
SPE	SWV	0.37 to 3.60	0.0086	[3]
GE<br>EPPG	LSV	0 to 7.50<br>0 to 6.00	0.805<br>0.267	[4]
PEDOT/β-CD/GCE	chronopotentiometry	0.10 to 100	0.0500	[5]
Fe^{3+}/p-a	DPV	0.10 to 6.5	0.0430	[6]
**Au/G-PLA**	**SWV**	**0.050 to 3.2**	**0.0160**	**This Work**

1. Hooshmand, S.; Es'haghi, Z. Microfabricated disposable nanosensor based on CdSe quantum dot/ionic liquid-mediated hollow fiber-pencil graphite electrode for simultaneous electrochemical quantification of uric acid and creatinine in human samples. *Anal. Chim. Acta* **2017**, *972*, 28–37, doi:10.1016/J.ACA.2017.04.035.
2. Kozitsina, A.; Shalygina, Z.; … S.D.-R.C.; 2009, undefined Catalytic systems based on the organic nickel (II) complexes in chronoamperometric determination of urea and creatinine. *Springer* **2009**, *58*, 1119–1125, doi:10.1007/s11172-009-0145-9.
3. Chen, J.C.; Kumar, A.S.; Chung, H.H.; Chien, S.H.; Kuo, M.C.; Zen, J.M. An enzymeless electrochemical sensor for the selective determination of creatinine in human urine. *Sensors Actuators, B Chem.* **2006**, *115*, 473–480, doi:10.1016/J.SNB.2005.10.015.
4. Randviir, E.P.; Kampouris, D.K.; Banks, C.E. An improved electrochemical creatinine detection method via a Jaffe-based procedure. *Analyst* **2013**, *138*, 6565–6572, doi:10.1039/C3AN01431B.
5. Naresh Kumar, T.; Ananthi, A.; Mathiyarasu, J.; Joseph, J.; Lakshminarasimha Phani, K.; Yegnaraman, V. Enzymeless creatinine estimation using poly(3,4-ethylenedioxythiophene) -β-cyclodextrin. *J. Electroanal. Chem.* **2011**, *661*, 303–308, doi:10.1016/J.JELECHEM.2011.08.001.
6. Fava, E.L.; Prado, T.M. do; Garcia-Filho, A.; Silva, T.A.; Cincotto, F.H.; Cruz de Moraes, F.; Faria, R.C.; Fatibello-Filho, O. Non-enzymatic electrochemical determination of creatinine using a novel screen-printed microcell. *Talanta* **2020**, *207*, 120277, doi:10.1016/j.talanta.2019.120277.

Table S7. Effect of possible interfering on the determination of CNN.

Interfering	Ratio (0.7:3.5 mmol)	Response (µA)	Response (%)
CNN	-	15.1	-
Glucose	1:5	14.3 ± 0.71	94.70 ± 4.97
AA	1:5	16.6 ± 1.13	109.9 ± 6.81
AU	1:5	13.8 ± 0.34	91.40 ± 2.46
Reduced glutathione	1:5	14.7 ± 0.24	97.30 ± 1.63

Table S8. Comparison between the proposed genosensor characteristics and works from the literature**.**

Electrode	Technique	Linear range (nmol L^{-1})	LOD (nmol L^{-1})	Ref.
3D-PP genosensor	DPV	10.0 to 500.0	15.0	[7]
probe-SH/ AuNTs /CSPE	DPV	-	0.000022	[8]
Biosensor	DPV	0.0001 to 3.0	0.000045	[9]
GONC platform	DPV	0.1 to 0.000001	186.0	[10]
FPCB	DPV	100 fg/mL to 1 µg /mL	33 fg/mL	[11]
label-free DNA capacitive biosensor	EIS	10.0 to 500.0	10.0	[12]
**Genosensor**	**CV**	**1000.0 to 50000.0**	**300.0**	**This Work**

7. Crevillen, A.G.; Mayorga-Martinez, C.C.; Vaghasiya, J. V.; Pumera, M. 3D-printed SARS-CoV-2 RNA genosensing microfluidic system. *Adv. Mater. Technol.* **2022**, doi:10.1002/ADMT.202101121.
8. del Caño, R.; García-Mendiola, T.; García-Nieto, D.; Álvaro, R.; Luna, M.; Iniesta, H.A.; Coloma, R.; Diaz, C.R.; Milán-Rois, P.; Castellanos, M.; et al. Amplification-free detection of SARS-CoV-2 using gold nanotriangles functionalized with oligonucleotides. *Microchim. Acta* **2022**, *189*, doi:10.1007/s00604-022-05272-y.
9. Deng, Y.; Peng, Y.; Wang, L.; Wang, M.; Zhou, T.; Xiang, L.; Li, J.; Yang, J.; Li, G. Target-triggered cascade signal amplification for sensitive electrochemical detection of SARS-CoV-2 with clinical application. *Anal. Chim. Acta* **2022**, *1208*, doi:10.1016/j.aca.2022.339846.
10. Ang, W.L.; Lim, R.R.X.; Ambrosi, A.; Bonanni, A. Rapid electrochemical detection of COVID-19 genomic

sequence with dual-function graphene nanocolloids based biosensor. *FlatChem* **2022**, *32*, doi:10.1016/j.flatc.2022.100336.
11. Damiati, S.; Sopstad, S.; Peacock, M.; Akhtar, A.S.; Pinto, I.; Soares, R.R.G.; Russom, A. Flex printed circuit board implemented graphene-based DNA aensor for setection of SARS-CoV-2. *IEEE Sens. J.* **2021**, *21*, 13060–13067, doi:10.1109/JSEN.2021.3068922.
12. Hwang, C.; Park, N.; Kim, E.S.; Kim, M.; Kim, S.D.; Park, S.; Kim, N.Y.; Kim, J.H. Ultra-fast and recyclable DNA biosensor for point-of-care detection of SARS-CoV-2 (COVID-19). *Biosens. Bioelectron.* **2021**, *185*, doi:10.1016/j.bios.2021.113177.

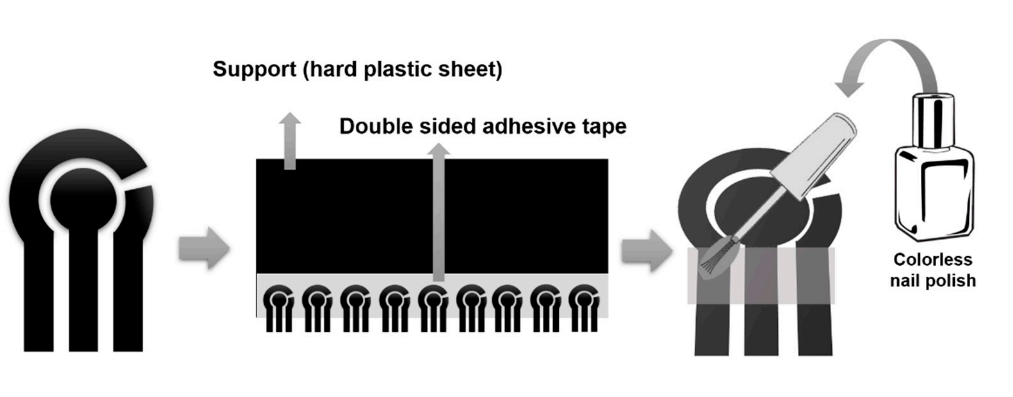

Scheme S1. Schematic illustration of the 3D printed electrodes assembly. Fixation on the hard plastic sheet support and delimitation of the area with colorless nail polish.

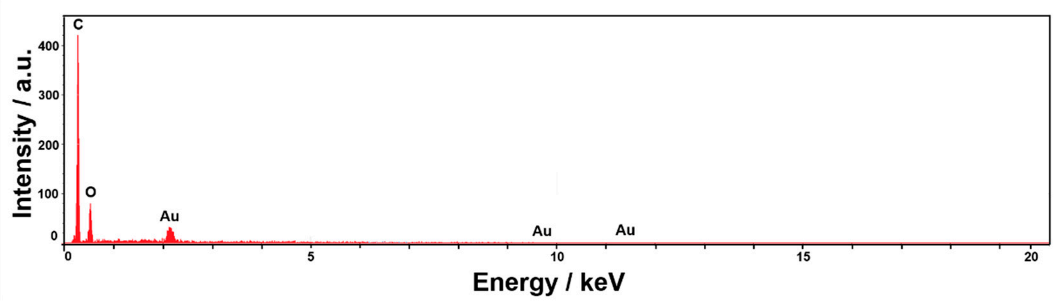

Figure S1. EDX spectrum for Au/G-PLA. The scale bar corresponds to 50µm.

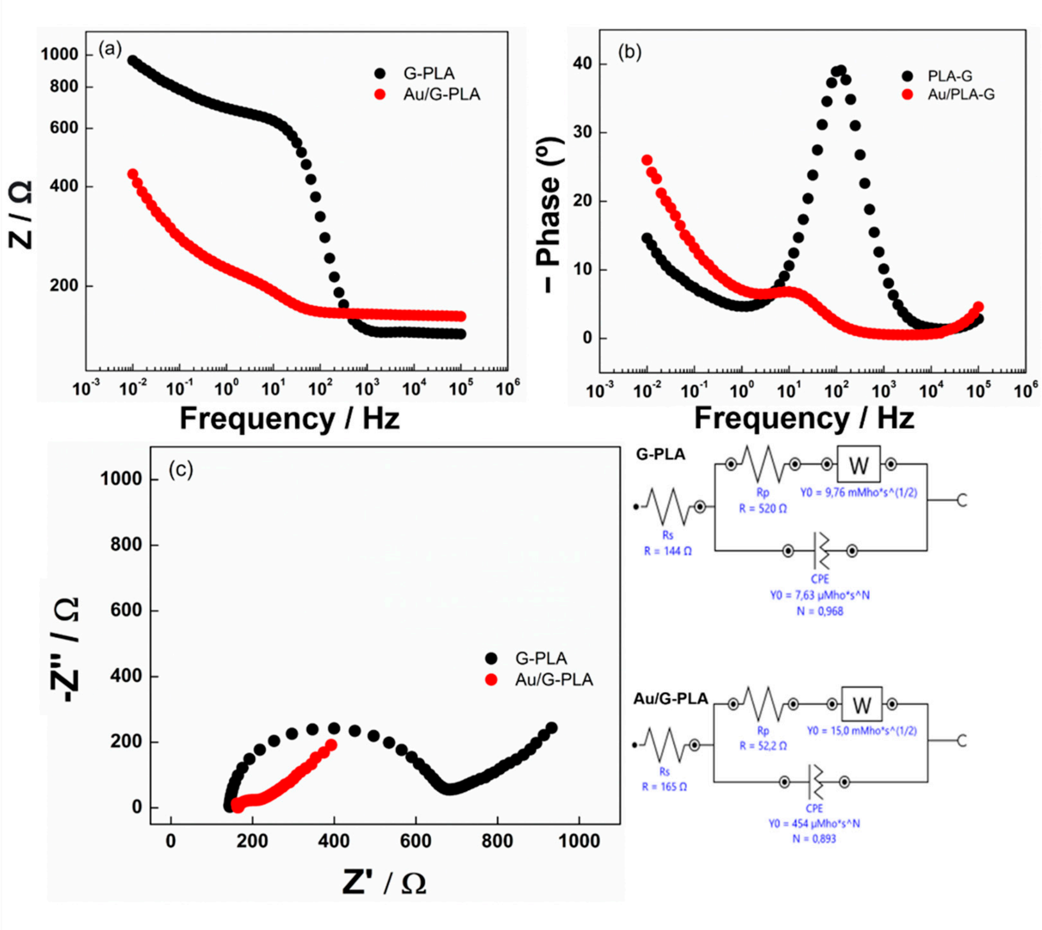

Figure S2. Impedance analyses of the G-PLA and Au/ G-PLA electrodes. (a) impedance magnitude and frequency correlation of the G-PLA (black line) and Au/ G-PLA (red line). (b) phase shift and frequency correlation of the G-PLA (black line) and Au/ G-PLA (red line). (c) Nyquist diagrams of the G-PLA (black line) and Au/ G-PLA (red line). Parameters: $E_{G\text{-}PLA}$ = 0.6 mV; $E_{Au/G\text{-}PLA}$ = 9.0 mV. Inset shows the respective equivalent circuits.

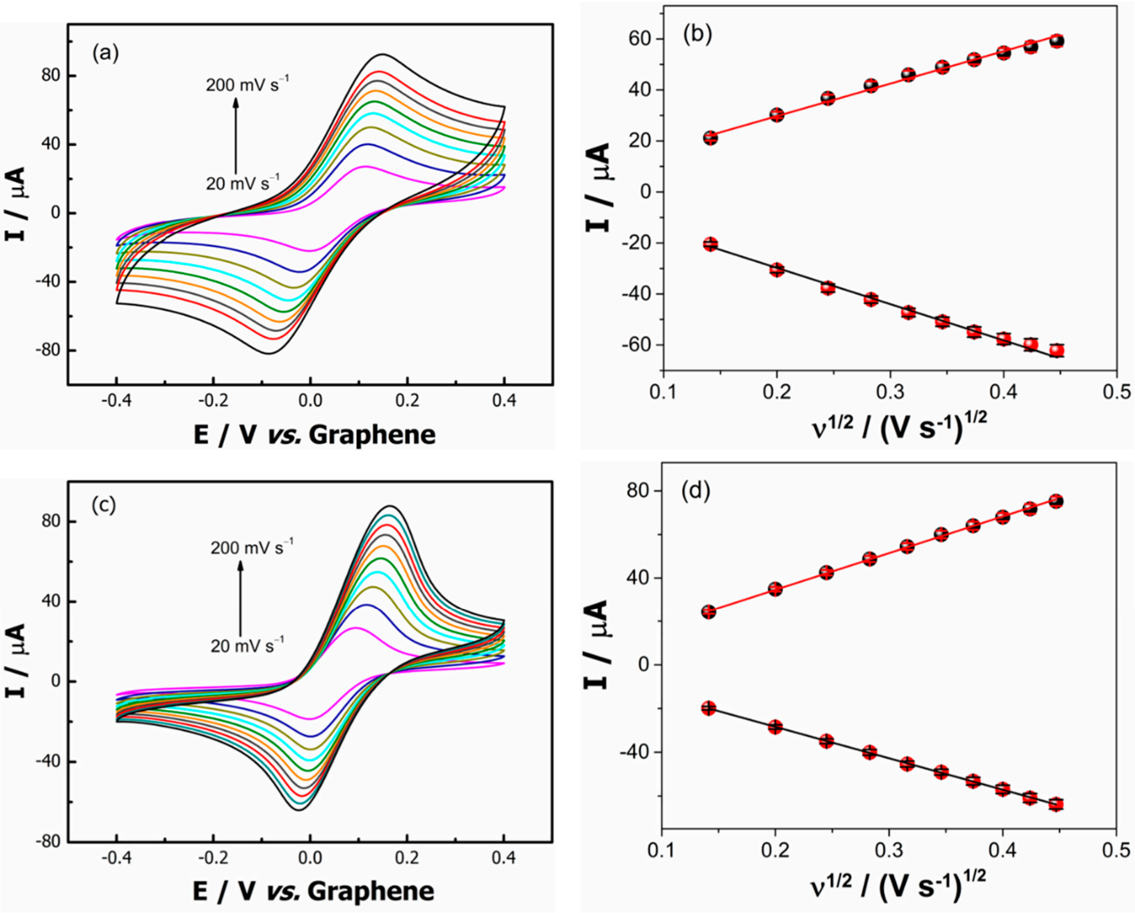

Figure S3. Cyclic voltammograms obtained for 1.0 mmol L^{-1} ferrocenemethanol in 0.1 mol L^{-1} KCl varying the scan rate in 20, 40, 60, 80, 100, 120, 140, 160, 180 and 200 mV s^{-1} using (a) G-PLA and (c) Au/G-PLA, and the respective plot of current response in function of $\upsilon^{1/2}$ for (b) G-PLA and (d) Au/G-PLA.

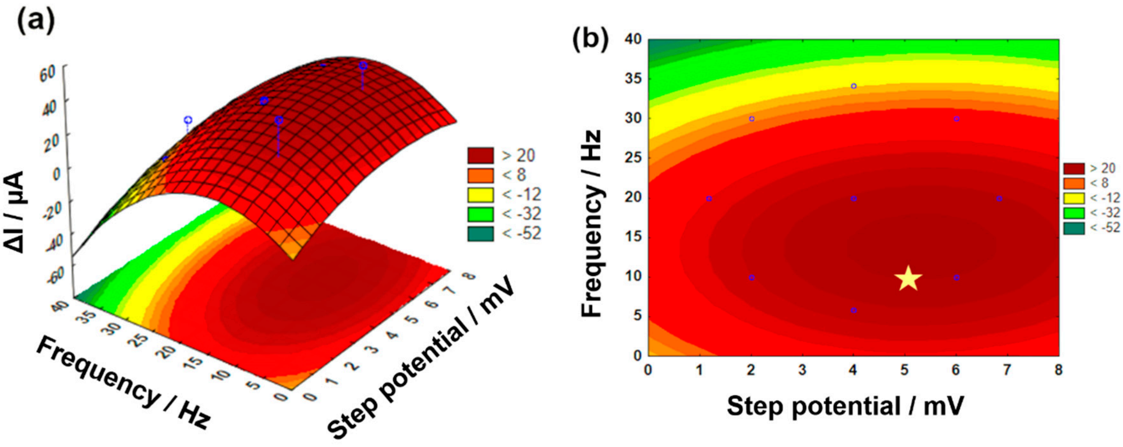

Figure S4. (a) Surface response and (b) level curve obtained for the optimization of the variables: step potential and frequency as a function of the current in the presence of 3.0 mmol L^{-1} CNN.

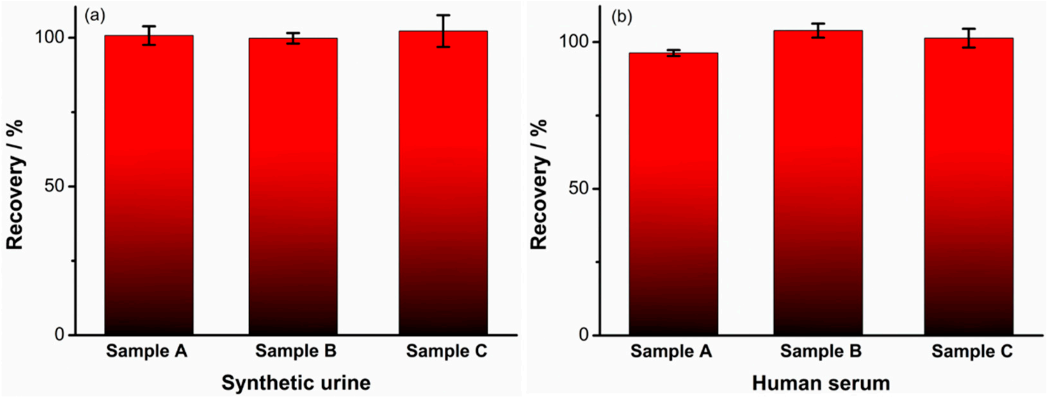

Figure S5. Recovery test performed from fortification of samples of (a) synthetic urine and (b) human serum with three known concentrations of CNN (0.1, 0.8 and 2.1 mmol L^{-1}).

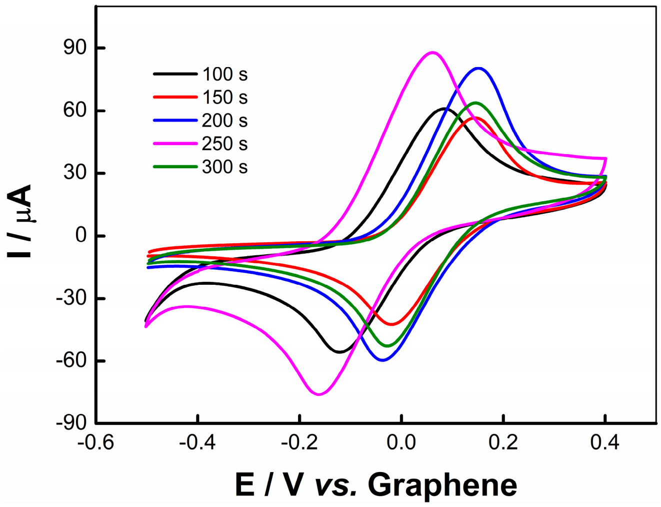

Figure S6. Cyclic voltammograms obtained after different gold deposition times on the G-PLA sensor. (a) (black line) 100 s, (red line) 150 s, (blue line) 200, (pink line) 250 and (green line) 300. All analyzes were carried out using 1 mmol L^{-1} ferrocenemethanol in 0.1 mol L^{-1} KCl with a scan rate of 50 mV s^{-1}.

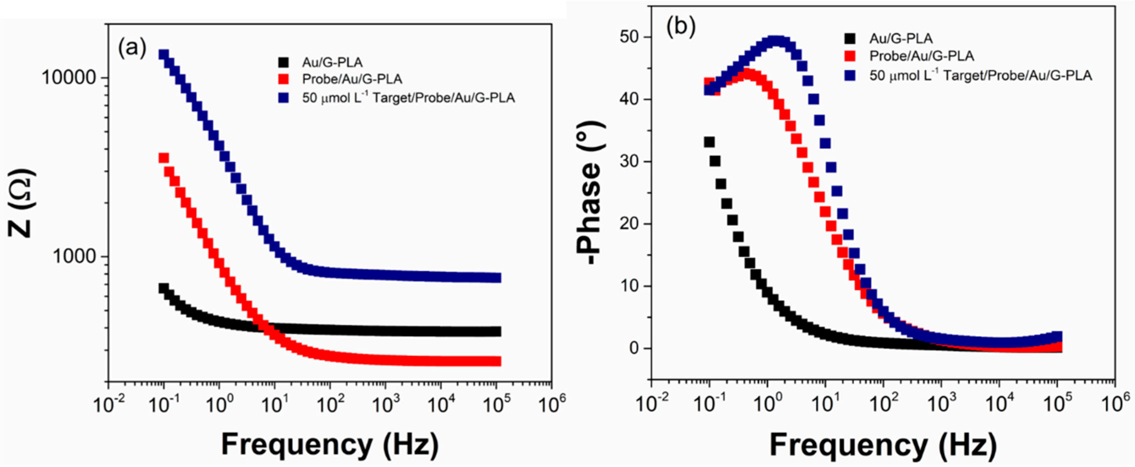

Figure S7. Impedance analysis of the (**black line**) Au/G-PLA, (**red line**) Probe/Au/G-PLA and (**blue line**) 50.0 µmol L^{-1} Target/Probe/Au/G-PLA. (a) impedance magnitude and frequency correlation. (b) phase shift and frequency correlation.

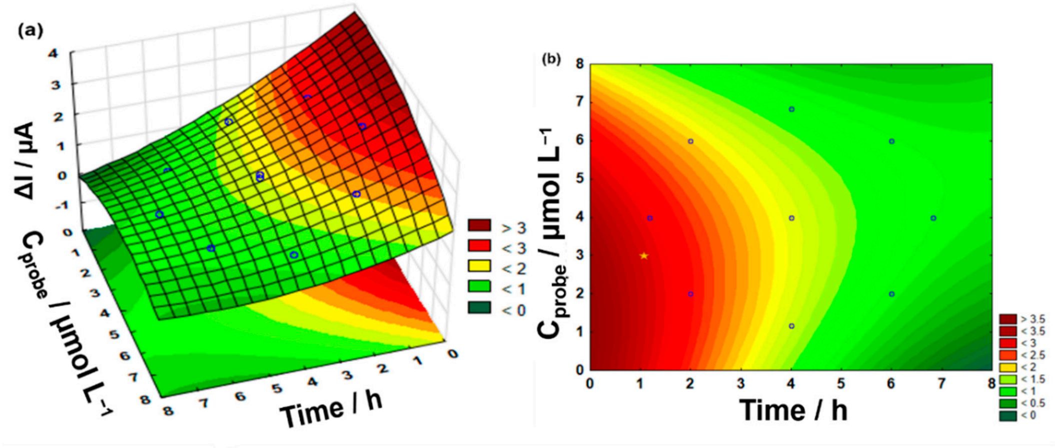

Figure S8. (a) Surface response and (b) level curve obtained for the optimization of the variables: concentration (µmol L^{-1}) and time (h) as a function of the current difference between the analytical signal of the sensor and the biosensor in presence of 1 mmol L^{-1} ferrocenemethanol in 0.1 mol L^{-1} KCl.

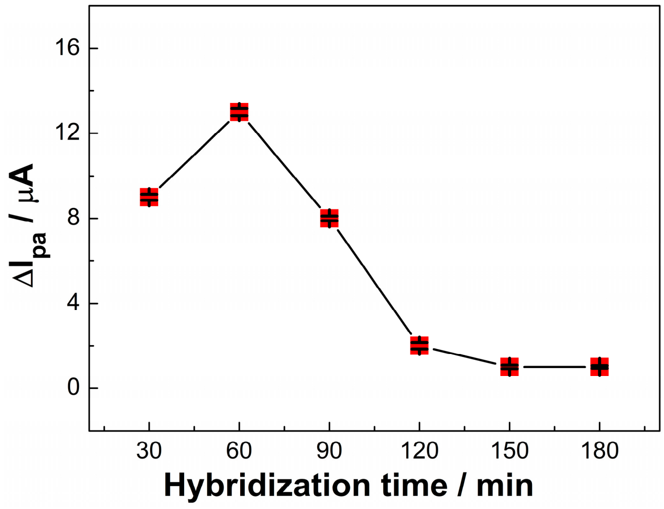

Figure S9. Responses obtained for different times (min) of hybridization of the target sequence with the biosensor. The response values were obtained as a function of the difference in the signal obtained by CV between the biosensor in the absence and the presence of 1.0 μmol L^{-1} of the target sequence using 1 mmol L^{-1} ferrocenemethanol in 0.1 mol L^{-1} KCl. CVs were carried out with a scan rate of 50 mV s^{-1}.

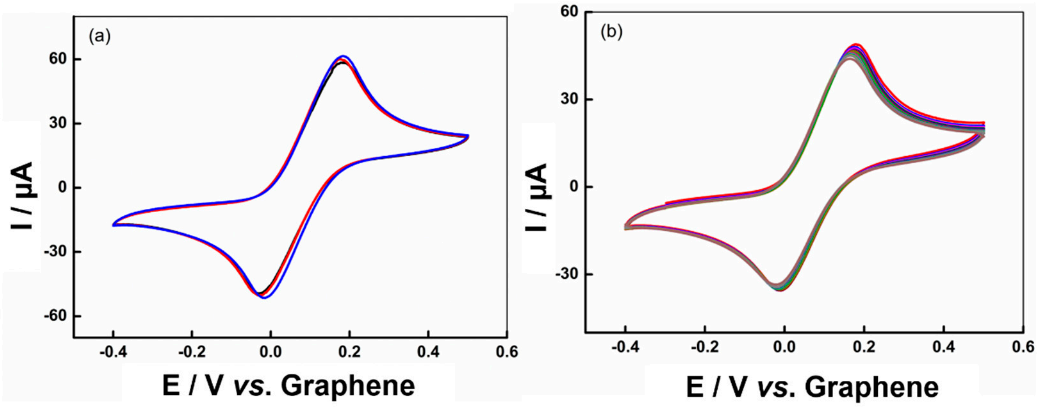

Figure S10. Cyclic voltammograms for (a) biosensor reproducibility (n = 3) and (b) repeatability (n = 20) in the presence of 1.0 mmol L^{-1} ferrocenemethanol in 0.1 mol L^{-1} KCl. Scan rate of 100 mV s^{-1}.

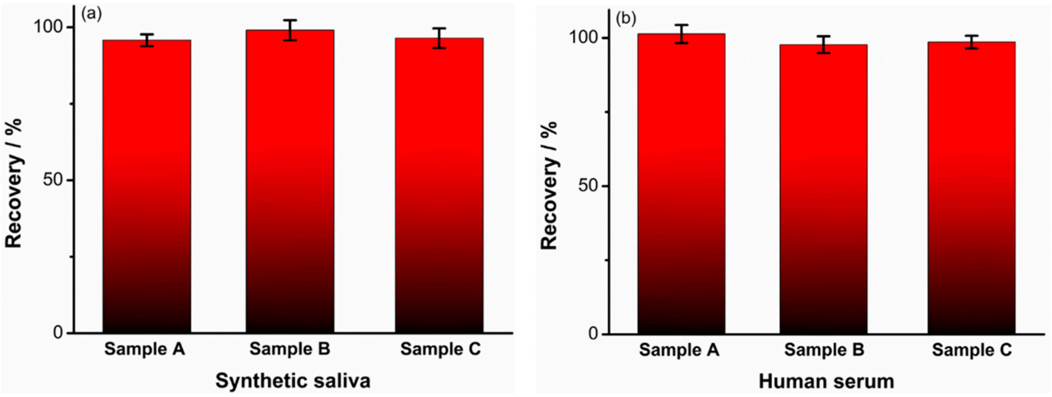

Figure S11. Recovery test performed from fortification of samples (a) synthetic urine and (b) human serum with three concentrations of Target (1.0, 25.0 and 50.0 μmol L−1).
